# Consecutive influenza surveillance of neuraminidase mutations and neuraminidase inhibitor resistance in Japan

**DOI:** 10.1111/irv.12624

**Published:** 2019-01-09

**Authors:** Yong Chong, Shinya Matsumoto, Dongchon Kang, Hideyuki Ikematsu

**Affiliations:** ^1^ Medicine and Biosystemic Science Kyushu University Graduate School of Medical Sciences Fukuoka Japan; ^2^ Department of Clinical Chemistry and Laboratory Medicine Kyushu University Hospital Fukuoka Japan; ^3^ Department of Clinical Chemistry and Laboratory Medicine Graduate School of Medical Sciences Kyushu University Fukuoka Japan; ^4^ Japan Physicians Association Influenza Study Group Tokyo Japan

**Keywords:** influenza, mutation, neuraminidase, neuraminidase inhibitor, resistance

## Abstract

**Background:**

The large consumption of neuraminidase inhibitors (NAIs) for the treatment of influenza virus infections places Japan at risk of becoming the epicenter of the global spread of NAI‐resistant viruses.

**Objective:**

To clarify NA amino acid mutations of epidemic influenza viruses in Japan and their related NAI resistance.

**Methods:**

A total of 1791 samples, including 396 A/H1N1pdm09, 1117 A/H3N2, and 278 B isolates, were collected to determine of their 50% inhibitory concentration (IC_50_) values by NAIs (oseltamivir, zanamivir, peramivir, and laninamivir) during the Japanese seasons from 2011‐2012 to 2016‐2017. Then, 380 samples including 49 A/H1N1pdm09, 251 A/H3N2, and 80 B isolates were sequenced for the entire NA genes.

**Results:**

Neuraminidase inhibitor‐resistant A/H1N1pdm09 viruses were detected at a frequency of 1.3% (5/396 isolates) in the epidemic seasons. None of the A/H3N2 and B viruses developed resistance to any of the four NAIs during the six seasons. Only five and 13 AA mutations were detected in the NA catalytic sites of A/H1N1pdm09 and A/H3N2 viruses, respectively. No mutations were observed in the catalytic sites of B viruses. Four of the five mutations in the catalytic sites of A/H1N1pdm09 consisted of H275Y, which was related to high resistance to oseltamivir and peramivir. Most (10/13) of the catalytic site mutations in A/H3N2 were associated with MDCK‐passaged induction (D151G/N). Finally, no mutations related to substantial NAI resistance were detected in the A/H3N2 and B viruses examined.

**Conclusion:**

These findings suggest that the NA catalytic sites of influenza viruses are well preserved. Even in Japan, no spread of NAI‐resistant viruses has been observed, and A/H1N1pdm09 viruses carrying H275Y remain limited.

## 1 INTRODUCTION

Seasonal influenza virus infections can cause high morbidity and mortality in humans, imposing a considerable socioeconomic burden. Although new functional drugs have been developed, the neuraminidase inhibitors (NAIs), including oral oseltamivir phosphate (Tamiflu^®^, oseltamivir), inhaled zanamivir hydrate (Relenza^®^, zanamivir), intravenous peramivir hydrate (Rapiacta^®^, peramivir), and inhaled long‐acting laninamivir octanoate hydrate (Inavir^®^, laninamivir), have been mainly used for the treatment of influenza virus infections. These NAIs exert their antiviral function by binding to the enzymatic catalytic sites of influenza surface protein NA.^1^ In the 2007‐2008, oseltamivir‐resistant seasonal A/H1N1 viruses carrying NA amino acid (AA) mutation H275Y rapidly spread throughout the world.^2^ We reported a prolonged duration of fever after administrating oseltamivir in patients infected with oseltamivir‐resistant A/H1N1 viruses during the 2008‐2009 season in Japan, which demonstrated the association between clinical effectiveness and the acquisition of resistance to anti‐influenza drugs.^3^, ^4^ In the subsequent seasons, oseltamivir‐resistant A/H1N1pdm09 viruses carrying H275Y were reported,^5^ causing a concern for worldwide spread.

A global surveillance system has been developed to detect NAI‐resistant influenza isolates and their related NA AA mutations.^6^ Currently, Japan is considered as the largest NAIs consuming country.^7^ Thus, Japan is in an environment where the emergence and selection pressure of NAI‐resistant influenza viruses are always strong compared with other countries and may become an epicenter of their global spread. Therefore, observing the transition of influenza NA AA mutations in Japan is considered important; however, comprehensive surveillance data, which include not only detecting NAI resistance and its related AA mutations but also determining NA AAs in epidemic viruses season by season, have not been fully reported in Japan. We have developed a network of physicians throughout Japan who routinely collect influenza virus samples, along with patient information.^3^, ^4^ In this study, we sequenced the full length of NA genes of epidemic influenza viruses isolated in Japan during the seasons from 2011‐2012 to 2016‐2017 to detect their AA mutations and examine its relationship with NAI resistance.

## 2 MATERIALS AND METHODS

### 2.1 Sample collection

As a part of a post‐marketing surveillance of laninamivir based on the rules of the Ministry of Health, Labor and Welfare of Japan, a series of surveillance studies have been conducted to examine the susceptibility (50% inhibitory concentration, IC_50_) of NAIs^8^, ^9^, ^10^, ^11^, ^12^; a total of 1791 samples, including 396 A/H1N1pdm09, 1117 A/H3N2, and 278 B isolates, were collected to determine their IC_50_ values during the seasons from 2011‐2012 to 2016‐2017 in Japan. As an additional part of our surveillance, a total of 380 samples, including 49 A/H1N1pdm09, 251 A/H3N2, and 80 B isolates, were analyzed to obtain NA sequencing data. Nasopharyngeal swabs for influenza virus isolation were collected, and the background history of patients who had a positive result on a rapid influenza antigen test, conducted in one of the member clinics of our nation‐wide study network of general practitioners, was determined. Informed consent was obtained from all patients. All study participants were outpatients, and this study did not include any patients with severe chronic respiratory diseases, renal diseases, liver diseases, or heart failure.

### 2.2 Influenza virus isolation and typing

Nasal aspirates, nasopharyngeal swabs, or self‐blown nasal discharge obtained from patients were soaked in virus transport medium and 75 μL of the medium was cultured using Madin‐Darby canine kidney (MDCK) cells. Monolayer‐cultured MDCK cells were inoculated with viruses collected from the clinical samples, and the cells were incubated at 34°C and 5% CO_2_. The wells were monitored daily until 7 days for virus growth by cytopathic effects, and the supernatant of the wells that exhibited sufficient cytopathic effects was collected. Viral RNA was extracted from infected MDCK cell culture supernatants using the Maxwell 16 LEV simply RNA Cells Kit (Promega, Madison, WI, USA). The A/H1N1pdm09, A/H3N2, and B subtypes were determined using type‐ and subtype‐specific primers.^13^

### 2.3 Measurement of virus susceptibility to NAIs

As a marker of virus susceptibility to NAIs, the IC_50_ values of oseltamivir, zanamivir, peramivir, and laninamivir were determined for each influenza isolate by a fluorescence‐based neuraminidase inhibition assay.^14^, ^15^ Reduced NAI susceptibility was defined according to the criteria provided by the World Health Organization.^16^ The IC_50_ value for each virus was compared with the median IC_50_ for the same subtype viruses isolated in the same season to calculate a fold increase. For A viruses, reduced inhibition (RI) was defined as a 10‐fold to 100‐fold increase in IC_50_ values, while highly reduced inhibition (HRI) was defined as more than 100‐fold increase in IC_50_ values. For B viruses, the corresponding fold increases of RI and HRI were 5‐50 and >50, respectively. Viruses exhibiting RI or HRI were considered as NAI resistant.

### 2.4 Sequencing

RT‐PCR was performed using the A/H1N1pdm09, A/H3N2, and B RNA samples.^17^, ^18^ After amplicon preparation, deep sequencing was conducted using the Illumina MiSeq sequencing system (Illumina, San Diego, CA, USA).^19^ Data processing was then performed using the pipeline prepared by Amelieff Co. (Tokyo, Japan). Finally, genome sequences were constructed using the reference sequences and filtered variants. The NA AA sequence was deduced from the obtained nucleotide sequence. In this study, a dominantly occupied nucleotide at each base position was determined as a consensus in the data processing. The NA AA lengths of A/H1N1pdm09, A/H3N2, and B viruses were 469, 469, and 466 AAs, respectively. The NA catalytic sites consisted of a total of 19 AAs, including AAs 118, 119, 151, 152, 156, 178, 179, 198, 222, 224, 227, 274, 276, 277, 292, 294, 371, 406, and 425 in N2 numbering. AA differences from consensus, which meant an AA type occupied by more than half isolates in the specific season, were defined as mutations. All AA mutations in NA and IC_50_ values in the corresponding isolates are shown in Tables S1–S13.

### 2.5 Nucleotide sequence accession number

The sequence data from this study were deposited into the DDBJ/EMBL/GenBank nucleotide sequence databases under the following accession numbers: LC406948‐LC407103, LC408965‐LC409058, and LC409129‐LC409257.

### 2.6 Statistical analysis

Categorical variables were analyzed using the chi‐square test. *P* value < 0.05 was considered to be statistically significant. All statistical analyses were performed using the jmp pro software, version 11 (SAS Institute, Inc., Cary, NC, USA).

## 3 RESULTS

The detection rates of circulating influenza A/H1N1pdm09, A/H3N2, and B viruses with reduced susceptibility (RI or HRI, RI/HRI) to NAIs during the seasons from 2011‐2012 to 2016‐2017 in Japan are shown in Table 1. A/H1N1pdm09 viruses displaying RI/HRI were detected at a frequency of 1.2% (2/172 isolates) and 1.4% (3/210 isolates) in the 2013‐2014 and 2015‐2016 epidemic seasons, respectively. Precisely, two viruses isolated in the 2013‐2014 season exhibited HRI by oseltamivir and RI by peramivir. Two of the three viruses isolated in the 2015‐2016 season displayed HRI by oseltamivir and RI by peramivir, and the remaining one showed RI only by oseltamivir. No A/H1N1pdm09 viruses exhibiting RI/HRI by zanamivir or laninamivir were detected during the seasons examined. For A/H3N2 and B, no virus with RI/HRI for any of the four NAIs was detected during the seasons from 2011‐2012 to 2016‐2017.

**Table 1:** Detection of reduced susceptibility by neuraminidase inhibitors (NAIs) in the circulating influenza viruses during the Japanese seasons from 2011‐2012 to 2016‐2017

Season	A/H1N1pdm09			A/H3N2			B		
	No. of samples	RI or HRI	Detection rate (%)	No. of samples	RI or HRI	Detection rate (%)	No. of samples	RI or HRI	Detection rate (%)
2011‐2012	0	NA	NA	283	0	0.0	42	0	0.0
2012‐2013	5	0	0.0	316	0	0.0	8	0	0.0
2013‐2014	172	2	1.2	49	0	0.0	106	0	0.0
2014‐2015	0	NA	NA	200	0	0.0	19	0	0.0
2015‐2016	210	3	1.4	20	0	0.0	82	0	0.0
2016‐2017	9	0	0.0	249	0	0.0	21	0	0.0
Total	396	5*	1.3	1117	0	0.0	278	0	0.0

HRI, highly reduced inhibition; NA, not available; RI, reduced inhibition.

RI or HRI was defined based on the criteria by the World Health Organization (Ref. 16).

*All the five A/H1N1pdm09 viruses displaying RI or HRI exhibited reduced inhibition by oseltamivir. Four of the five A/H1N1pdm09 viruses displaying RI or HRI exhibited reduced inhibition by peramivir.

NA AA mutation rates were compared between catalytic and non‐catalytic sites of each virus (Table 2). Only five AA mutations were detected in the catalytic sites of A/H1N1pdm09 viruses, although their AA mutation rates at catalytic sites were not significantly different from those at non‐catalytic sites (0.54% vs 0.45%). For A/H3N2, only 13 AA mutations were detected in the catalytic sites. In the entire seasons examined, the AA mutation rates of A/H3N2 viruses were significantly lower at catalytic sites than at non‐catalytic sites (0.27% vs 0.65%, *P *=* *0.001). No AA mutations were detected in the catalytic sites of B viruses, resulting in a significant lower frequency at the catalytic sites compared with those at the non‐catalytic sites (0.0% vs 0.70%, *P *=* *0.001). In the entire season examined, the AA mutations per sample at the catalytic sites of A/H1N1pdm09, A/H3N2, and B were 0.10, 0.05, and 0.00 AAs, respectively, which confirmed the extremely low number in the AA mutations at the NA catalytic sites (Table S14).

**Table 2:** Comparison of amino acid mutation rates between catalytic and non‐catalytic sites of neuraminidase genes

Virus	Season	No. of samples	All sites			Catalytic sites			Non‐catalytic sites			
			No. of total AA mutations	No. of total AAs	Mutation rate (%)	No. of total AA mutations	No. of total AAs	Mutation rate (%)	No. of total AA mutations	No. of total AAs	Mutation rate (%)	*P* value
A/H1N1pdm09	2013‐2014	20	49	9380	0.52	2	380	0.53	47	9000	0.52	0.991
	2015‐2016	20	42	9380	0.45	3	380	0.79	39	9000	0.43	0.308
	2016‐2017	9	14	4221	0.33	0	171	0.00	14	4050	0.35	0.441
	Total	49	105	22 981	0.46	5	931	0.54	100	22 050	0.45	0.711
A/H3N2	2011‐2012	48	120	22 512	0.53	3	912	0.33	117	21 600	0.54	0.387
	2012‐2013	48	97	22 512	0.43	4	912	0.44	93	21 600	0.43	0.971
	2013‐2014	20	77	9380	0.82	1	380	0.26	76	9000	0.84	0.219
	2014‐2015	40	165	18 760	0.88	0	760	0.00	165	18 000	0.92	0.008
	2015‐2016	19	58	8911	0.65	1	361	0.28	57	8550	0.67	0.367
	2016‐2017	76	226	35 644	0.63	4	1444	0.28	222	34 200	0.65	0.081
	Total	251	743	117 719	0.63	13	4769	0.27	730	112 950	0.65	0.001
B	2013‐2014	20	82	9320	0.88	0	380	0.00	82	8940	0.92	0.061
	2014‐2015	19	41	8854	0.46	0	361	0.00	41	8493	0.48	0.186
	2015‐2016	20	29	9320	0.31	0	380	0.00	29	8940	0.32	0.266
	2016‐2017	21	98	9786	1.00	0	399	0.00	98	9387	1.04	0.040
	Total	80	250	37 280	0.67	0	1520	0.00	250	35 760	0.70	0.001

AA, amino acid.

The neuraminidase AA lengths of A/H1N1pdm09, A/H3N2, and B viruses were 469, 469, and 466 AAs, respectively. The neuraminidase catalytic sites consisted of a total of 19 AAs.

Based on the reports on NA AA mutations associated with reduced inhibition by NAIs,^2^, ^6^, ^20^ RI/HRI‐related AA mutations were extracted from NA sequencing data examined in this study (Table 3). Most (10/13 mutations) of the AA mutations detected in the catalytic sites of A/H3N2 viruses were D151G/N mutations, which were found to be induced by an MDCK cultivation.^21^, ^22^ T148I in the non‐catalytic sites was also reported to be associated with an MDCK passage.^23^ These AA mutations were not detected in any of unpassaged clinical samples.^22^ Finally, few AA mutations (3/4769, 0.06%, referred to Table 2) in the catalytic sites of A/H3N2 viruses occurred without their D151G/N mutations. With regard to the substantial RI/HRI‐related AA mutations detected in this study, four A/H1N1pdm09 viruses displaying HRI by oseltamivir and RI by peramivir contained H275Y mutations. The isolate exhibiting RI by oseltamivir in the 2015‐2016 season did not carry any of the RI‐related AA mutations (DS6‐349 in Table S8). D199N in A/H1N1pdm09, D151E, K249E, G320E, and S331R in A/H3N2, and I262T in B were extracted as RI/HRI‐related AA mutations, based on their related references^6^, ^24^, ^25^, ^26^; however, no virus harboring these AA mutations showed substantial RI/HRI based on their IC_50_ values.

**Table 3:** Extraction of NA amino acid mutations associated with reduced susceptibility by NAIs

Virus	Season	Sample	AA mutation	Catalytic site	IC_50_ (nmol/L)				
					Oseltamivir	Peramivir	Zanamivir	Laninamivir	Reference
Culture‐induced mutation									
A/H3N2	2011‐2012	DS2‐35	T148I		0.5	0.6	1.4	2.0	23
		DS2‐44	T148I		0.5	0.6	1.3	1.9	
		DS2‐113	T148I		0.9	1.0	2.2	3.8	
		DS2‐209	T148I		0.5	0.6	1.7	1.8	
		DS2‐393	T148I		0.8	0.8	2.1	3.4	
		DS2‐516	T148I		0.5	0.8	2.3	1.8	
		DS2‐94	T148I		0.3	0.3	1.0	1.2	
		DS2‐415	D151G	Catalytic site	0.8	0.8	1.9	2.9	22
		DS2‐94	D151N	Catalytic site	0.3	0.3	1.0	1.2	22
	2012‐2013	DS3‐34	T148I		0.4	0.8	1.0	2.9	
		DS3‐229	T148I		0.8	0.6	1.7	2.8	
		DS3‐94	T148I		0.9	1.0	2.3	4.0	
		DS3‐4	D151N	Catalytic site	1.0	0.8	2.4	3.0	
		DS3‐51	D151N	Catalytic site	0.6	0.7	1.1	3.9	
		DS3‐122	D151N	Catalytic site	0.8	0.8	2.3	4.4	
		DS3‐360	D151N	Catalytic site	0.9	0.7	2.1	3.3	
	2013‐2014	DS4‐9	T148I		0.7	0.6	1.9	2.2	
		DS4‐354	D151N	Catalytic site	0.8	0.8	2.4	2.4	
	2014‐2015	None							
	2015‐2016	DS6‐47	D151G	Catalytic site	1.0	0.8	2.1	4.5	
	2016‐2017	DS7‐298	D151G	Catalytic site	0.9	0.6	1.9	4.8	
		DS7‐6	D151G	Catalytic site	0.5	0.4	1.6	1.6	
Drug resistance‐related mutation									
A/H1N1pdm09	2013‐2014	DS4‐549	H275Y	Catalytic site	150.0	19.0	1.0	2.2	5
		DS4‐371	H275Y	Catalytic site	130.0	12.0	0.8	2.3	
	2015‐2016	DS6‐15	D199N	Catalytic site	1.2	0.6	2.5	2.4	24
		DS6‐352	H275Y	Catalytic site	150.0	10.0	1.3	3.9	
		DS6‐528	H275Y	Catalytic site	130.0	17.0	1.2	2.7	
	2016‐2017	None							
A/H3N2	2011‐2012	None							
	2012‐2013	DS3‐369	K249E		1.2	0.9	2.9	3.6	6
		DS3‐173	S331R		1.2	0.7	2.7	3.9	26
	2013‐2014	None							
	2014‐2015	DS5‐388	G320E		0.9	0.6	2.2	2.8	26
	2015‐2016	DS6‐11	S331R		0.8	0.3	2.3	2.9	
	2016‐2017	DS7‐129	D151E	Catalytic site	1.1	0.8	2.3	4.8	25
B	2013‐2014	None							
	2014‐2015	None							
	2015‐2016	None							
	2016‐2017	DS7‐219	I262T		14	2.1	7.1	16	6

AA, amino acid; HRI, highly reduced inhibition; IC_50_, 50% inhibitory concentration; NA, neuraminidase; NAIs, neuraminidase inhibitors; RI, reduced inhibition.

RI/HRI‐related AA mutations were extracted from NA sequencing data examined in this study, based on the previous reports.

The AA position numberings of A/H1N1pdm09, A/H3N2, and B viruses are based on N1, N2, and B type‐specific numbering, respectively.

Median IC_50_ values and fold changes are shown in Tables S1‐S13.

## 4 DISCUSSION

In this study, the detection rates of NAI‐resistant influenza viruses and their related NA AA mutations were analyzed from comprehensive NA sequencing data of epidemic isolates in Japan, where NAIs are massively used. Consequently, NA AA mutations associated with NAI resistance were only H275Y of H1N1pdm09. The frequency of H275Y detected in Japan was approximately 1%, which was similar to that reported by a global surveillance.^6^ For A/H3N2 and B, no virus exhibiting reduced inhibition by NAIs was detected in Japan. These results were also similar to those reported by a global surveillance, which indicated that NAI‐resistant A/H3N2 and B viruses were detected at a lower frequency (<1.0%) compared with NAI‐resistant A/H1N1pdm09 viruses.^6^ This study showed that few AA mutations in the catalytic sites of A/H3N2 viruses were found without their D151G/N mutations. Our findings suggested that the AA mutations in the NA catalytic sites were far more difficult to occur than those in the non‐catalytic sites, at least for A/H3N2 and B viruses. The acquisition of NAI resistance‐related mutations in epidemic influenza viruses does not seem to occur easily, even in Japan.

In this study, the detection rates of AA mutations in the non‐catalytic sites of NA genes were also examined, and relatively many mutations to the catalytic sites were detected. In addition, the AA mutations associated with reduced inhibition by NAIs, which were indicated by the previous reports, were also included in their mutations. However, these presumed NAI resistance‐related mutations did not show any substantial reduced inhibition in this study (Table 3). These findings suggested that the direct contribution of mutations in the NA non‐catalytic sites to NAI resistance was extremely rare.

This study showed that oseltamivir‐resistant A/H1N1pdm09 viruses carrying H275Y did not spread throughout Japan. The sporadic community spread of the H275Y‐resistant viruses was reported in Newcastle, Australia, in 2011 and in Sapporo, Japan, in 2013.^27^, ^28^ These viruses discovered in the two cities carried additional AA mutations, V241I and N369K, which were presumed to confer a positive effect on viral fitness.^29^ These viruses were also shown to have carried the third additional AA mutation, N386S/K, which was in contrast suggested to cause a negative effect on viral fitness in the presence of the V241I and N369K mutations.^29^, ^30^ In our data (Table S15), both N386 and K386 in addition to I241 and K369 were detected in the 2013‐2014 season; in contrast, only K386 together with I241 and K369 was detected in the subsequent seasons. The two oseltamivir‐resistant H275Y viruses isolated in the 2015‐2016 season contained V241I, N369K, and N386K. The dissemination of the H275Y‐resistant viruses may remain restricted, due to impaired viral fitness mechanism that is caused by additional mutations.

Our study does not demonstrate whether NA AA mutations determined in the analysis are substantial in the circulating viruses or induced in the process of cultivation, because a direct NA comparison was not conducted between clinical samples and their MDCK‐passaged influenza viruses. However, it is highly possible that T148I and D151G/N are AA mutations induced by an MDCK passage, based on their previous reports,^22^, ^23^ although these mutations did not cause a substantial increase in their IC_50_ values (Table 3).

The mechanism of acquiring drug resistance in NA catalytic sites for zanamivir and laninamivir is different from that for oseltamivir and peramivir, due to a difference in their side chain structures.^31^ Laninamivir is licensed only in Japan and has been used increasingly in the last few years, being the main NAI used. No mutations associated with laninamivir resistance were detected in our study. This finding also suggests that the NA catalytic sites of influenza viruses are well preserved. Even in Japan, the largest consumer of NAIs, no spread of NAI‐resistant viruses with mutations related to clinical ineffectiveness, such as a prolonged duration of fever, has been observed, and the emergence of A/H1N1pdm09 viruses carrying H275Y remains limited. NAIs are currently effective for the treatment of influenza virus infections, although a continued surveillance is warranted in the future.

## CONFLICT OF INTEREST

This investigation was partially supported by Daiichi Sankyo Co., Ltd. H. Ikematsu has previously received honoraria from Daiichi Sankyo Co., Ltd, for medical advice.
